# Activation of the neurokinin-1 receptor by substance P triggers the release of substance P from cultured adult rat dorsal root ganglion neurons

**DOI:** 10.1186/1744-8069-3-42

**Published:** 2007-12-25

**Authors:** He-Bin Tang, Yu-Sang Li, Koji Arihiro, Yoshihiro Nakata

**Affiliations:** 1 Department of Pharmacology, Graduate School of Biomedical Sciences, Hiroshima University, Kasumi 1-2-3, Minami-ku, Hiroshima 734-8553, Japan; 2 Department of Anatomical Pathology, Hiroshima University Hospital, Kasumi 1-2-3, Minami-ku, Hiroshima 734-8551, Japan

## Abstract

**Background:**

Although substance P (SP) is an important primary afferent modulator in nociceptive processes, it is unclear whether SP regulates its own release from primary sensory neurons.

**Results:**

Using a highly sensitive radioimmunoassay for SP, we have demonstrated that the activation of neurokinin-1 receptor by SP or GR73632 (a potent neurokinin-1 receptor agonist) triggered an increase of SP release from cultured adult rat dorsal root ganglion (DRG) neurons depending on the dose and exposure time within 60 min, and thereafter, the SP release level gradually decreased over 360 min. Accompanying the SP release, a significant reduction in the percentage of neurons expressing neurokinin-1 receptor on their membranes during exposure to SP (200 pg/dish) occurred time dependently (56 ± 5% and 32 ± 2% at 180 and 360 min, respectively). The GR73632-evoked (10 nM, 60 min) SP release was attenuated by several inhibitors for mitogen-activated protein kinase kinase, p38 mitogen-activated protein (MAP) kinase and cyclooxygenase-2 (COX-2), protein kinase C (PKC), respectively. In contrast, a c-Jun NH_2_-terminal kinase inhibitor increased the GR73632-evoked SP release.

**Conclusion:**

These results indicate that the neurokinin-1 receptor activation by its agonists regulates the SP release process involving the activation of MAP kinases, PKCs and COX-2 from cultured DRG neurons.

## Background

Substance P (SP) is one member of the tachykinin neuropeptide family that shares a carboxy-terminal sequence Phe-X-Gly-Leu-Met-NH_2 _[1], along with neurokinin A, neurokinin B and neuropeptide K, neuropeptide-γ. SP is derived from the preprotachykinin-A gene, and is synthesized in the dorsal root ganglion (DRG) neurons [2]. SP is released through a very complex process involving some important intracellular effectors, such as extracellular calcium influx, 1,4,5-inositol trisphosphate-induced calcium release, the activation of extracellular signal-regulated kinase (ERK), cyclooxygenases (COXs) and prostaglandins, and the cyclic AMP-dependent protein kinase A (PKA) from primary afferent neurons to convey information about various noxious stimuli [3-6]. Previous studies have demonstrated that SP functions as an important neurotransmitter and/or, as a primary afferent modulator in nociceptive processes, thereby potentiating excitatory input to nociceptive neurons [7-10].

The biological effects of SP are mediated through binding to the specific G-protein-coupled neurokinin receptors designated neurokinin-1, -2 and -3 receptors [11]. Once activated by SP, the neurokinin receptor induces the activation of several second messenger systems, such as phospholipase C (PLC) and adenylate cyclase, thereby increasing the consequent production of 1,4,5-inositol trisphosphate and cyclic AMP [12]. Moreover SP has been shown to induce the activation of ERK1/2 and p38 mitogen-activated protein (MAP) kinases, nuclear factor-kappa B and protein kinase C (PKC), and thereafter to increase the production of prostaglandin E_2 _and the expression of COX-2 [13-15]. Interestingly, both anatomical and functional evidence have also suggested that neurokinin-1 receptors may function as auto-receptors in DRG neurons [16,17]. In view of the above-mentioned observations on the release and the biological effects of SP, it is considered important to clarify whether the release of SP is induced via the activation of neurokinin-1 receptor, while also elucidating what type of signaling can occur in the process of SP release via the neurokinin-1 receptor from cultured adult rat DRG neurons. Hence, the objective of the present study is designed to demonstrate whether the release of SP may be stimulated by itself through the activation of its receptors and the involvement of some important intracellular effectors (such as MAP kinase, PLC and PKC, COX and PKA) from cultured DRG neurons.

## Results

### The release of SP induced by itself from cultured rat DRG neurons

**Figure 1:**
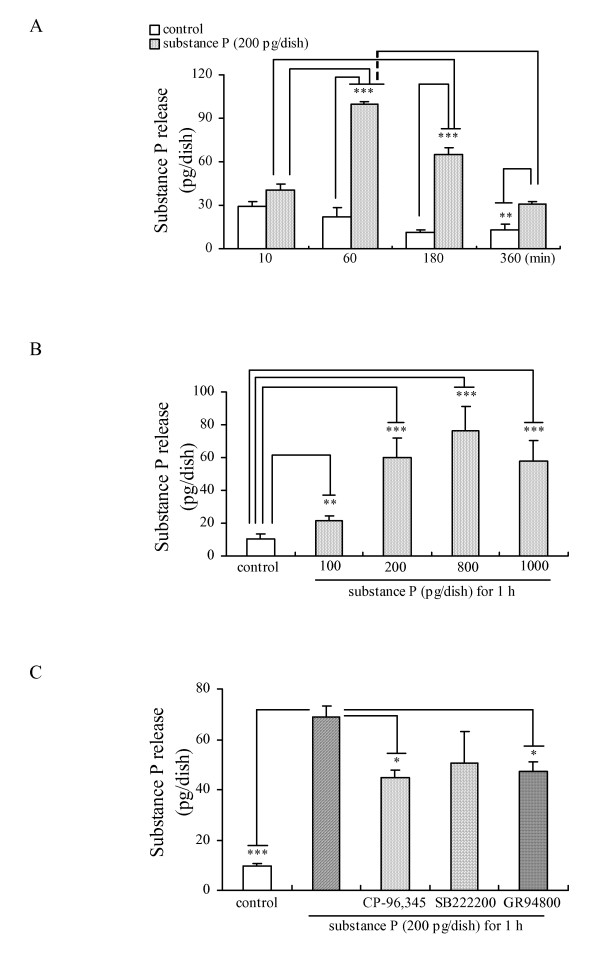
**The SP release induced by itself from cultured adult rat DRG neurons**. Time-dependent (A) and dose-dependent (B) effects of SP on its own release from cultured DRG neurons. (C) Effects of neurokinin receptor antagonists (1 μM CP-96,345, 1 μM SB222200 and 100 nM GR94800) on the SP release from cultured DRG neurons exposed to SP. The data are expressed as means ± S.E.M. (bars) from 3–5 (A), 4 (B) or 3 (C) separate experiments. *, ** and *** denote *p *< 0.05, 0.01 and 0.001, respectively.

To investigate whether SP induces its own release from cultured DRG neurons, we examined the effects of SP on the release of SP in a dose- and time-dependent manner. Based on the amount of the SP release induced by various chemicals in our previous study [5,6,18], we selected 200 pg/dish of SP as an appropriate concentration for our experimental conditions for investigating the possibility of self-induced SP release. A time-course of SP release induced by SP (200 pg/dish) from cultured DRG neurons is shown in Fig. 1A. As a peak of SP release was observed after the 60 min incubation, we decided to use the 60 min incubation with SP (200 pg/dish) as an experimental condition for examining various drugs on the self-induced SP release. As shown in Fig. 1B, SP evoked a dose-dependent release of SP during a 60 min incubation of cultured DRG neurons.

It is well known that all three neurokinin receptors (neurokinin-1, -2 and -3 receptors) are expressed in DRG neurons. We therefore investigated whether neurokinin-1 and/or other neurokinin receptor(s) are involved in the SP release induced by itself. The increase in the SP release evoked by itself was partially significantly attenuated by 1 μM CP-96,345 (a selective antagonist of neurokinin-1 receptor) [19] and by 100 nM GR94800 (a selective antagonist of neurokinin-2 receptor) [20], not by 1 μM SB222200 (a selective antagonist of neurokinin-3 receptor) [20] as shown in Fig. 1C, whereas these antagonists did not have any effect when used alone (Data not shown). Based on the results shown in Fig. 1C, both the neurokinin-1 and -2 receptors seem to be involved in the process of SP release, however, the detailed pharmacological action(s) of the neurokinin-2 receptor in the substance P release will be examined in future experiments.

### Immunocytochemical localization of the neurokinin-1 receptor and SP

**Figure 2:**
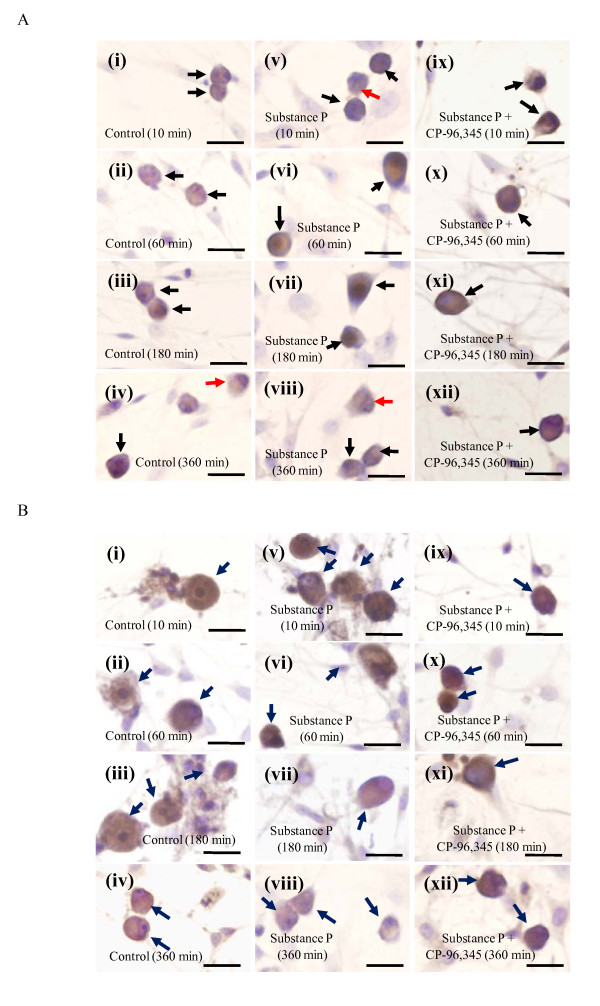
**The immunocytochemical localization of the neurokinin-1 receptor and SP in cultured adult rat DRG cells**. The time-dependent expression of the neurokinin-1 receptor (A) and SP (B) in cultured DRG neurons divided into three groups: control group (i-iv), 200 pg/dish SP group (v-viii) and 200 pg/dish SP plus 1 μM CP-96,345 group (ix-xii). Photomicrographs were taken with the Olympus IX71 inverted microscope (x40). The arrows indicate the neurokinin-1 receptor- or SP-positive neurons (brown). The red arrows indicate the DRG neurons where the neurokinin-1 receptors are not expressed in their membrane. Scale bars: 20 μm.

We next investigated the time-dependent changes in the expression of SP and its neurokinin-1 receptor in cultured DRG cells incubated in serum-free DMEM (containing peptidase inhibitors) with or without SP (200 pg/dish) in the presence/absence of 1 μM CP-96,345. The specific type of cells indicated by arrows in Figs 2A and 2B can easily be identified to be DRG neurons by the morphology (characteristic phase bright cell bodies bearing either bi- or multi-polar neurites observed by using the Olympus IX71 inverted microscope with a 20X Ph1 objective) and by the Schwann cell marker S100 (Rabbit anti-cow S100; 1:1,000 dilution; Dako, Glostrup, Denmark)- and the astrocyte marker GFAP (Mouse anti-glial fibrillary acidic protein; 1:800 dilution; Chemicon, Temecula, CA)-negative expression (Data not shown). As shown in Figs 2Ai–iv, the neurokinin-1 receptors were distributed on the membrane and in the cytoplasm of smaller DRG neurons in a naive state. The ratio of the number of neurons expressing the neurokinin-1 receptor on their membranes to the total number of neurokinin-1 receptor-positive neurons in a randomly selected field in each image from three separate experiments was simultaneously calculated. The percentage of neurons expressing neurokinin-1 receptor on their membrane in a naive state was 91 ± 5%, 82 ± 4% and 74 ± 4%, 71 ± 3% in Figs 2Ai–iv, respectively. Interestingly, a significant reduction in the percentage of neurons expressing neurokinin-1 receptor on their membranes was observed after the time-dependent stimulation of 200 pg/dish SP (85 ± 4%, 71 ± 7% and 56 ± 5%, 32 ± 2% in Figs 2Av–viii, respectively) in comparison to their respective controls (*p *> 0.05, *p *> 0.05 and *p *< 0.05, *p *< 0.001, respectively). Furthermore, the reduction induced by SP was attenuated by the pretreatment (10 min) with 1 μM CP-96,345 (86 ± 4%, 85 ± 7% and 84 ± 2%, 84 ± 5% in Figs 2Aix–xii, respectively). On the other hand, we observed the SP content to be mainly distributed in the cytoplasm of the smaller DRG neurons (Fig. 2B). The long-term (60 to 360 min) exposure of the DRG neurons to SP (200 pg/dish) thus resulted in a reduction of the SP content in their cytoplasm (Figs 2Bv–viii), whereas the pretreatment with 1 μM CP-96,345 blocked the reduction of the SP content (Figs 2Bix–xii). The SP-evoked expression change of the neurokinin-1 receptor from the membrane to the intracellular compartment in somas of DRG neurons should therefore be considered as the receptor internalization (a pharmacologically specific index of neurokinin-1 receptor-SP interaction).

### The SP-induced changes of neurokinin-1 receptor expression in the cytosolic and membrane fractions

**Figure 3:**
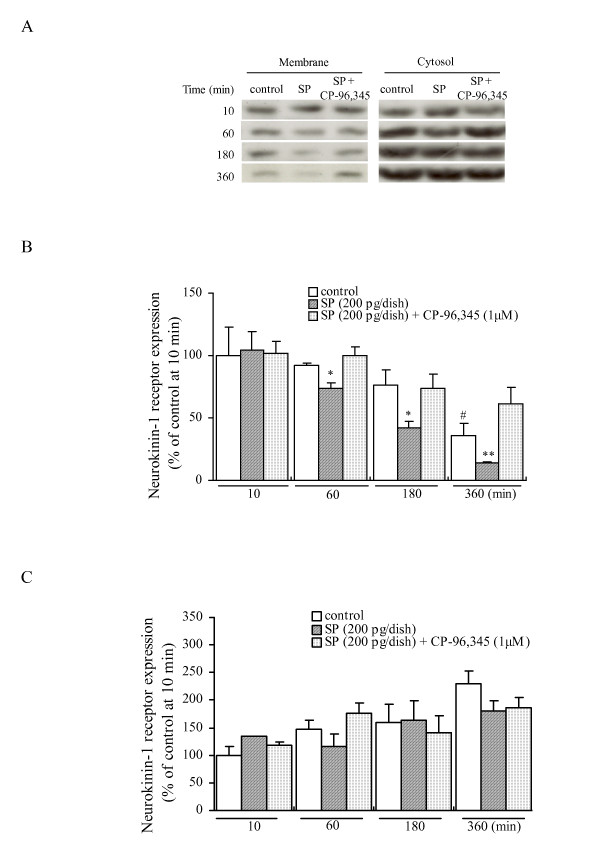
**Time-course studies of the SP-induced neurokinin-1 receptor expression in the cytosolic and membrane fractions from cultured adult rat DRG neurons**. (A) Representative blots of the neurokinin-1 receptor expression in the cytosolic and membrane fractions from cultured DRG neurons exposed to SP for 10 to 360 min. All data have been quantified by normalizing the neurokinin-1 receptor expression level of control in the membrane (B) or cytosolic (C) fraction at 10 min, respectively. The data are expressed as the means ± S.E.M. (bars) from 3 separate experiments. # denotes a *p *< 0.05 as compared to the effect of untreated control at 10 min, *p < 0.05 and **p < 0.01 versus the effect of the untreated control at the same time by a one-way analysis of variance followed by Bonferroni's test.

Based on the neurokinin-1 receptor localization results shown in Fig. 2A, we attempted to further quantify the levels of neurokinin-1 receptor in the cytosolic and membrane fractions of cultured DRG neurons. As shown in Figs 3A and 3B, the time-dependent (10–360 min) exposure of cultured DRG neurons to SP (200 pg/dish) resulted in a significant decrease of the neurokinin-1 receptor expression in the membrane fractions (104 ± 15%, 74 ± 4% and 42 ± 5%, 14 ± 1% in Figs 3A and 3B, respectively, compared with the control at 10 min). The SP-induced decrease of that was completely blocked by the 10 min pretreatment with 1 μM CP-96,345 (102 ± 9%, 100 ± 7% and 74 ± 11%, 61 ± 14% in Figs 3A and 3B, respectively, compared with the control at 10 min). Therefore the activation of neurokinin-1 receptor by SP is accompanied by the internalization of its receptors, however, we could not identify the proteins detected by anti-substance P receptor antibody in the cytosol as a functional neurokinin-1 receptor. Interestingly, we observed a gradual decrease of the neurokinin-1 receptor expression in the membrane fraction from the untreated DRG neurons (the control). These data suggest the endogenous SP released from the DRG neurons could enhance the turnover of neurokinin-1 receptor from the membrane to the cytosol as the released SP is not taken into the DRG cells. However, after the induction of internalization by the stimulation of SP, the level of the cytosolic proteins detected by anti-substance P receptor antibody does not change, thus suggesting a part of internalized neurokinin-1 receptor protein could be degraded to maintain the amount of neurokinin-1 receptor to some extent within the cytosol.

### Characteristics of the GR73632-induced release of SP

**Figure 4:**
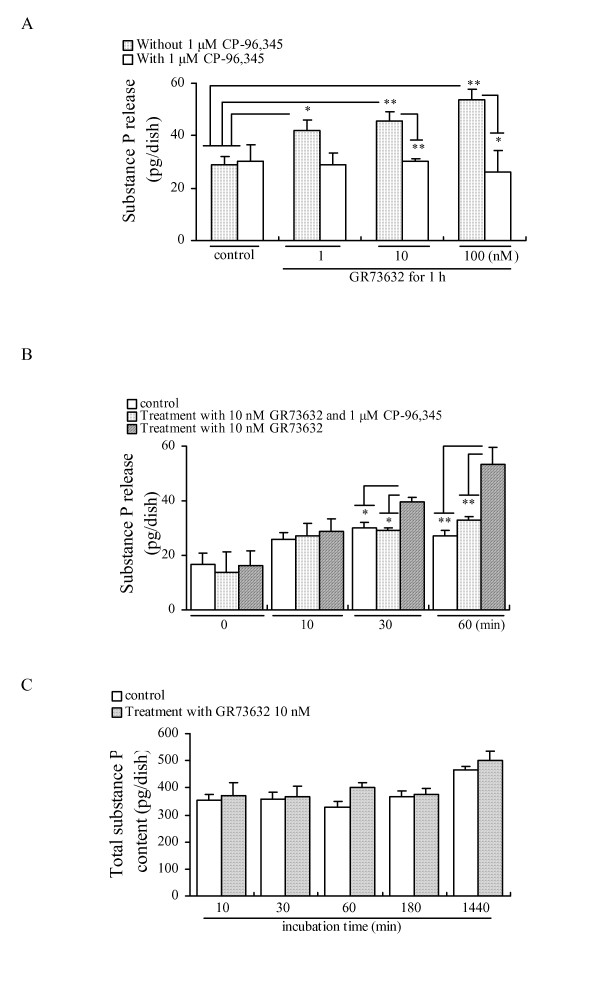
**Effects of GR73632 on the release and total content of SP from cultured adult rat DRG neurons**. Dose-dependent (A) and time-dependent (B) effects of GR73632 on the release of SP from cultured adult rat DRG neurons. (C) Total SP content from cultured DRG neurons and the culture medium. The data are expressed as means ± S.E.M. (bars) from 3–4 (A), 4 (B) or 3 (C) separate experiments. * and ** denote *p *< 0.05 and 0.01, respectively.

The data shown in Figs 1C and 2 indicate that the activation of neurokinin-1 receptor is involved in the SP release induced by SP. We therefore selected another potent neurokinin-1 receptor agonist GR73632 (showing no cross-reactivity with anti-SP serum in radioimmunoassay) to further investigate the molecular mechanisms of the SP release via the activation of the neurokinin-1 receptor in cultured DRG neurons. As shown in Fig. 4A, it was observed that a 60 min incubation with GR73632 (1–100 nM) stimulated a significant increase in the SP release in a dose-dependent manner from the cultured DRG neurons. The increases in the SP release induced by GR73632 at various concentrations were almost completely blocked by the 10 min pretreatment with CP-96,345. Based on the results shown in Fig. 4B, we thought that the 60 min incubation with 10 nM GR73632 was an appropriate condition in our experiments. However, we observed that a time-dependent treatment (10 min to 24 h) of GR73632 (10 nM) did not cause any detectable change in the total amount of SP content from cultured DRG neurons and the culture medium (Fig. 4C). In addition, significant changes of the preprotachykinin mRNA expression were not caused by the time-dependent (10–360 min) exposure of cultured DRG neurons to 10 nM GR73632 (Data not shown). Therefore, the effect of GR73632 under our experimental conditions should not be considered to cause a rapid increase in the synthesis of the SP content in cultured DRG neurons.

**Figure 5:**
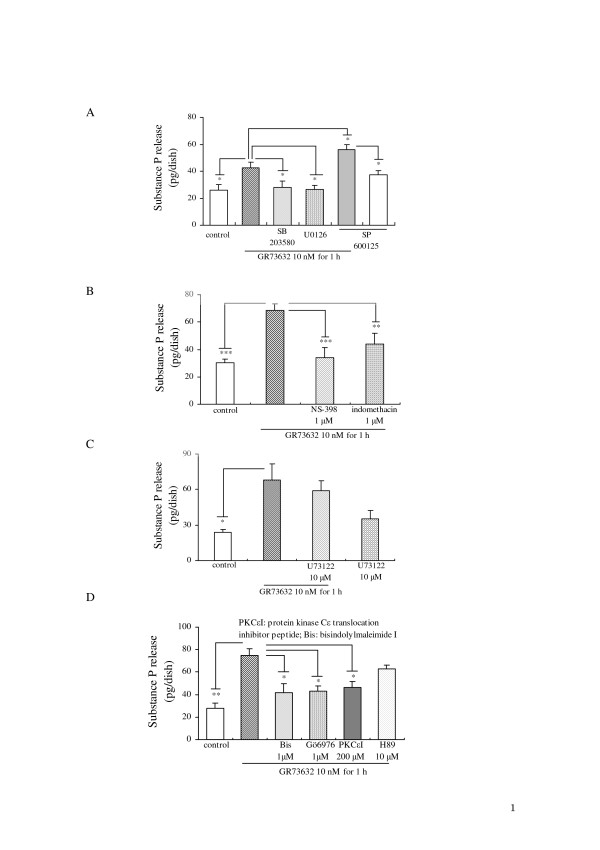
**Characteristics of the GR73632-evoked SP release from cultured adult rat DRG neurons**. Some cells were left untreated as a control, all other cells were treated with GR73632 alone or together with three MAP kinases cascades inhibitors (10 μM U0126, 15 μM SB203580 and 30 μM SP600125), PLC inhibitor (10 μM U73122) or with COX inhibitors (1 μM NS-398 and 1 μM indomethacin), PKC inhibitors (1 μM Gö6976, 1 μM bisindolymaleimide, 200 μM PKCε translocation inhibitor peptide) or with PKA inhibitor (10 μM H89) in DMEM (serum free) for 60 min. The data are expressed as the means ± S.E.M. (bars) from 3–5 (A), 6 (B) or 4 (C), 4 (D) separate experiments. *, ** and *** denote *p *< 0.05, 0.01 and 0.001, respectively.

Whether the complex process of SP release induced by GR73632 requires the activation of MAP kinases, COXs, PLC or PKCs, PKA in cultured DRG neurons, still remains to be elucidated. As shown in Fig. 5A, both U0126 and SB203580 exhibited a significant inhibitory effect on the SP release evoked by GR73632, whereas SP600125 stimulated an increase in the SP release. When these three inhibitors [6] of MAP kinases were used alone, only a c-Jun NH_2_-terminal kinase (JNK) inhibitor SP600125 had a weak tendency to increase the SP release (Fig. 5A). We also observed that the GR73632-induced SP release (Figs 5B and 5D) was significantly attenuated by NS-398 (a highly selective inhibitor of COX-2) [5], indomethacin (a non-selective inhibitor of COX1/2) [6] or by PKCε translocation inhibitor peptide [21], Gö6976 (a selective inhibitor for PKCα-, βI-isozymes) [22] or bisindolylmaleimide I (a broad inhibitor for PKCα-, βI-, βII-, γ-, δ-, ε-isozymes) [22]. However, neither U73122 (a selective inhibitor of PLC) [23] nor H89 (a selective inhibitor of PKA) [4] influenced the SP release induced by GR73632 (Figs 5C and 5D).

### Up-regulation of COX-2 expression induced by SP and by GR73632

**Figure 6:**
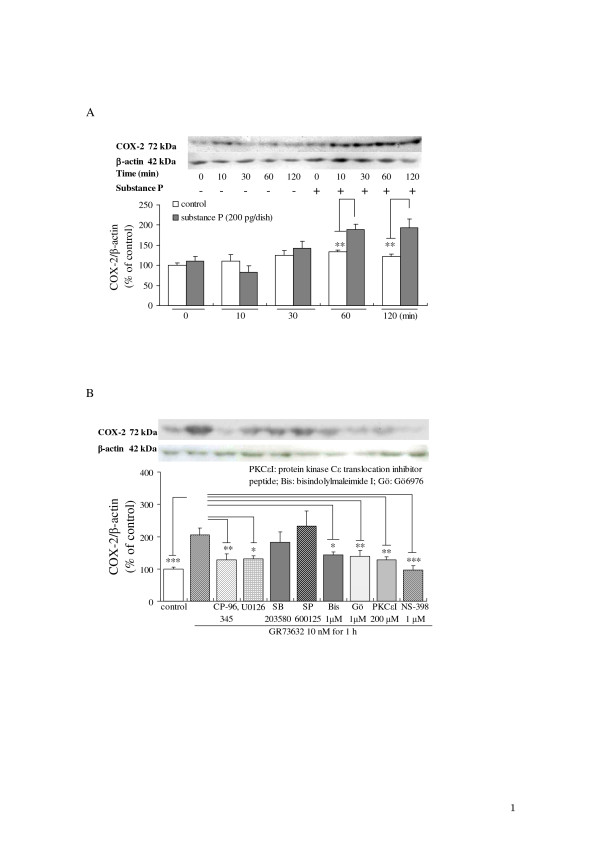
**Effects of SP and GR73632 on COX-2 protein expression in cultured adult rat DRG neurons**. (A) The time-dependence of COX-2 expression in cultured DRG neurons exposed to SP. (B) The protein levels of COX-2 in DRG neurons treated with GR73632, either alone or together with 1 μM CP-96,345, three MAP kinases cascades inhibitors (10 μM U0126, 15 μM SB203580 and 30 μM SP600125) or with 1 μM NS-398, PKC inhibitors (1 μM Gö6976, 1 μM bisindolymaleimide, 200 μM PKCε translocation inhibitor peptide) for 60 min were measured by Western blot analysis. All data have been normalized to the COX-2 expression level of control group. The data are expressed as the means ± S.E.M. (bars) from 4 (A) or 4 (B) separate experiments. *, ** and *** denote *p *< 0.05, 0.01 and 0.001, respectively.

To clarify the possible signal transduction pathway(s) involved in the SP release via the activation of neurokinin-1 receptor, the activation status of COX-2 was assessed using specific antibodies for COX-2 after the stimulation of SP or GR73632 in the absence or presence of various inhibitors. The time-dependent exposure of DRG neurons to SP (200 pg/dish) resulted in the significant increase of de novo protein synthesis of COX-2 (Fig. 6A). The 60 min incubation with 10 nM GR73632 also up-regulated the expression of COX-2 protein (Fig. 6B), whereas the increase in the expression of COX-2 protein evoked by GR73632 was significantly attenuated by the pretreatment with CP-96,345, U0126, NS-398 or three inhibitors for PKC isozymes (Gö6976, bisindolylmaleimide I and PKCε translocation inhibitor peptide), respectively. However, neither SB203580 nor SP600125 influenced the up-regulation of COX-2 expression induced by GR73632.

## Discussion

In the present study, we demonstrated for the first time that the activation of neurokinin-1 receptor by its agonists (SP or GR73632) modulates the SP release from cultured DRG neurons through some important intracellular effectors.

During the 360 min exposure of DRG to SP (200 pg/dish), a peak response in the SP release was observed within the first 60 min, whereas a gradual decrease in the SP release level was obtained at later time points (180 and 360 min) (Fig. 1A). The release pattern of SP induced by itself may be explained by the internalization and recycling of the neurokinin-1 receptor [24], because the immunocytochemical and Western blotting results (Figs 2A and 3) showed the existence of neurokinin-1 receptor internalization induced by SP, and it also indicated an inhibitory effect of the continuous exposure to SP on the neurokinin-1 receptor recycling. In addition, the time-dependent reduction in SP content of the DRG neurons exposed to SP provides an explanation for the existence of SP release (Fig. 2B). Our present findings are therefore in agreement with the hypothesis that SP induces its own release via its auto-receptor, the neurokinin-1 receptor [16,17]. Our data also indicated that SP may function as a neuromodulator in the slow release response itself from cultured DRG neurons. The precise mechanism of the association between the SP release and the neurokinin-1 receptor internalization should be revealed by further studies.

The neurokinin-1 receptor has a preferential affinity for SP [25]. The expression of the neurokinin-1 receptor is observed mainly in the small rat DRG neurons by in situ hybridization [26]. We have therefore focused our attention on the involvement of the neurokinin-1 receptor in the SP release from cultured DRG neurons. GR73632 (a selective agonist of the neurokinin-1 receptor) stimulated a significant increase in the release of SP via acting on the neurokinin-1 receptor. To clarify the characteristics of SP release via the neurokinin-1 receptor, we further investigated the possible involvement of several intracellular effectors, such as MAPKs, COX-2 and PLC, PKA and PKCs.

The MAPKs family contains at least three protein kinases in series: JNK, p38 MAP kinase and MEK (a kinase immediately upstream of ERK that phosphorylates the tyrosine and threonine residues on ERK1/2 required for activation). They are often involved in the intracellular transmission of extracellular signals [27]. In our previous study, the activation of ERK1/2 was demonstrated to be involved in the SP release evoked by bradykinin [6]. Fiebich *et al*. [13] and Yang *et al*. [28] also indicated that ERK1/2 and p38 MAP kinase can be rapidly activated by SP in a dose-dependent manner. In view of the above-mentioned observations and the results shown in Fig. 5A, p38 MAP kinase and MEK seem to play a role in increasing the release of SP. In contrast, the data shown in Fig. 5A suggest that the JNK is likely to be associated with the suppression of SP release from cultured DRG neurons, although this kinase was reported to function as an important factor involved in SP-stimulated secretion and production of inflammatory mediators in rat peritoneal mast cells [29].

It is well known that the binding of the ligand to the neurokinin-1 receptor activates several second messenger systems, including 1,4,5-inositol trisphosphate formation via PLC activation and cyclic AMP accumulation via adenylate cyclase [12]. The activation of cyclic AMP-dependent PKA was also reported to be involved in the SP release caused by prostaglandin E_2_[30]. However, we observed that PLC and PKA did not influence the SP release via the neurokinin-1 receptor from cultured DRG neurons.

PKC is a family of serine- and threonine-specific protein kinases, which has been suggested to function as an important intracellular signaling molecule in primary afferent nociceptors, while also being implicated in acute and chronic inflammatory as well as neuropathic pain. The activation of PKC was also reported to induce the synthesis of COX-2 and the release of prostaglandin E_2 _in primary midbrain astrocytes [31]. Previous study in our laboratory [32] has shown that the time-dependent and transient induction of COX-2 mRNA was observed 30 min after bradykinin (a potent pro-inflammatory mediator) stimulation in cultured DRG neurons. The short-term exposure of the DRG neurons to bradykinin at 1 μM for 30 min also induced small but significant amounts of prostaglandin E_2 _release depending on the activation of COX1/2. Our present findings also demonstrated a significant increase in COX-2 expression stimulated during a 60 min exposure of cultured DRG neurons to SP (Fig. 6A). Moreover, the de novo protein synthesis of COX-2 requires the activation of PKCs and MEK (Fig. 6B). In view of the above-mentioned observations and results shown in Figs 5D and 6, it is suggested that PKC isozymes including ε type play the important roles in the de novo protein synthesis of COX-2 via the neurokinin-1 receptor, and thereby increase the SP release from cultured DRG neurons.

Interestingly, our results in the present work are partially consistent with several previous observations *in vivo*. For example, the activation of neurokinin-1 receptors by intrathecal injection of SP evokes thermal hyperalgesia and spinal prostaglandin E_2 _release which can be reversed by spinal COX-2 inhibition and by the intrathecal delivery of the p38 MAP kinase inhibitor SB203580; spinal PKC inhibition blocks the intrathecal injection of SP-mediated thermal hyperalgesia [34-37]. Moreover, the inhibition of PLC-β and PKC-ε can completely block both the neurokinin-1 receptor agonist-induced TRPV1 (transient receptor potential vanilloid subtype 1) potentiation and heat hyperalgesia [38]. Similar to the observation reported by Zhang et al. [38], we also observed that the activation of neurokinin-1 receptor by its agonist GR73632 to enhance the capsaicin-evoked substance P release in our latest research, which thus demonstrated that the potentiation of capsaicin-evoked substance P release by GR73632 via the activation of neurokinin-1 receptor depends on the activation of PKCs, MEK and p38 MAP kinase, PLC and COXs from cultured DRG neurons (Unpublished data). However, the detailed relationships among the activation of PLC, PKC, MAP kinases and COXs regarding the enhancement of capsaicin-evoked substance P release by GR73632 via the activation of neurokinin-1 receptor will be described in a study to be published in the not-so-distant future. Based on our findings and the above-mentioned observations reported previously, we proposed a possible molecular mechanism underlying the SP release induced by the neurokinin-1 receptor agonists (SP and GR73632) from cultured rat DRG neurons. The long-term exposure of DRG neurons to SP or GR73632 resulted in the activation of MEK, p38 MAP kinase and PKC at an early stage and thereafter induced the synthesis of COX-2, which they contribute to the SP release triggered by the neurokinin-1 receptor.

## Conclusion

This study demonstrated that the activation of neurokinin-1 receptor by its agonists (SP and GR73632) regulates the SP release process depending on the activation of MEK, p38 MAP kinase and PKC, and the de novo protein synthesis of COX-2, while also indicating that the JNK likely has an inhibitory effect on the SP release. These observations provide important evidence to help us understand the molecular mechanisms of inflammatory pain modulated by SP in primary afferent neurons.

## Methods

### Isolation and culture of rat DRG cells

According to a previously described method [5,6,23], DRGs of young adult Wistar rats (6–9 weeks of age) were dissociated into single isolated neurons and non-neuronal cells by the treatment of collagenase (Sigma, St. Louis, MO, USA) and trypsin (Invitrogen, Burlington, ON, Canada). The cells (3 DRGs/dish) were maintained at 37°C in a water-saturated atmosphere with 5% CO_2 _for 5 days before the initiation of the experiments. All procedures for animal experiments were performed according to the Guide for Animal Experimentation, Hiroshima University, and the Committee of Research Facilities for Laboratory Animal Sciences, Graduate School of Biomedical Sciences, Hiroshima University, Japan.

### Measurement of SP content in the culture medium and in the cultured rat DRG neurons

Except for some cultured cells treated by peptidase inhibitors containing 1 μM phosphoramidon (Sigma), 4 μg/ml bacitracin (Sigma) and 1 μM captopril (Sigma) alone (as a control), other cultured cells were exposed to SP (Peptide Institute, Osaka, Japan) or to GR73632 (Sigma), either alone or together with various inhibitors such as Gö6976 (Calbiochem, Darmstadt, Germany), PKCε translocation inhibitor peptide (Calbiochem) and bisindolylmaleimide I (Calbiochem), indomethacin (Sigma) and SB222200 (Sigma), GR94800 (Sigma) and U73122 (Sigma), SP600125 (Sigma) and H89 (Seikagaku, Tokyo, Japan) in 1 ml serum-free DMEM (Nissui, Tokyo, Japan) containing peptidase inhibitors for a designated period of time at 37°C in a water-saturated atmosphere with 5% CO_2_. Thereafter, the SP content collected from the culture medium and the cultured DRG neurons was measured by a highly sensitive radioimmunoassay [5,18], respectively. For examining the amount of SP-induced SP release in the present experiments, we developed a new computational method. Briefly, SP at a specified concentration (100, 200 and 800, 1,000 pg/dish) was used to stimulate two groups of cultured DRG neurons in both the absence and presence of various antagonists for three neurokinin receptors (neurokinin-1, -2 and -3 receptors). The SP content was immediately collected from the culture medium (1 ml) after the SP stimulation for the first group, and the amount of SP content was examined from the culture medium (1 ml) after the SP stimulation lasted for 10, 60, 180, 360 minutes, respectively, for the second group. The numerical difference in the SP content between the two groups is considered to be the amount of SP release induced by this specified concentration of SP during a specific time period from cultured DRG neurons.

### Immunocytochemical staining for the neurokinin-1 receptor and SP in the cultured rat DRG neurons

Immunocytochemical staining for the neurokinin-1 receptor and SP in cultured DRG neurons on coverglasses was performed with a standard immunoperoxidase technique [Histofine Simple Stain Rat MAX-PO (MULTI) kit; Nichirei, Tokyo, Japan] according to the manufacturer's instructions. Briefly, 4% paraformaldehyde-fixed cultured DRG cells on coverglasses were incubated with anti-neurokinin-1 receptor (1:2,000 dilution; Sigma) or anti-SP serum (1:1,000 dilution; a gift of Dr. J.S. Hong, National Institute of Environmental Health Sciences, NIH, USA) [33]. After the treatment with Histofine simple stain rat MAX-PO (MULTI), color development (brown) was performed using a DAB substrate kit (Nichirei), and the coverglasses were counterstained with hematoxylin (blue). According to the manufacturer's instructions for the datasheet of anti-substance P receptor antibody (S8305, Sigma), it is guaranteed that the antibody specifically recognizes the neurokinin-1 receptor peptide (rat, amino acids 393–407) in immunoblotting. Immunocytochemical controls demonstrating antibody specificity for the neurokinin-1 receptor and SP included immunostaining cultured cells on coverglasses, but the primary antibody was omitted. The omission of the primary antibody resulted in no staining in the cells.

### Subcellular fractionation

After a 10 min pretreatment with the presence or absence of 1 μM CP-96,345 (Pfizer, Groton, CT, USA), the cultured DRG cells were incubated in serum-free DMEM (containing peptidase inhibitors) with or without SP (200 pg/dish) for 10, 60, 180, 360 minutes, respectively. The isolation of cytosolic and membrane fractions from these DRG cells was performed with a standard cell compartment kit fractionation procedure (Cell compartment kit; Qiagen, Tokyo, Japan) according to the manufacturer's instructions. Protein concentrations were determined, and then the neurokinin-1 receptor levels in the same amounts of cytosolic and membrane proteins were analyzed separately by a Western blot analysis.

### Western blot analysis

At the end of the SP release experiments, the cell samples were processed for Western blot analysis as previously described [6]. Primary antibodies were raised against COX-2 (1:1,000 dilution; COX-2 polyclonal antibody; Cayman Chemical, Ann Arbor, MI), β-actin (1:5,000 dilution; the mouse monoclonal antibody for β-actin; Sigma) or anti-substance P receptor (1:2,000 dilution; Sigma). The horseradish peroxidase-conjugated anti-rabbit and anti-mouse secondary antibodies (1:2,000 dilution; Cell Signaling Technology, Beverly, MA) were used for chemiluminescence detection according to the manufacturer's instructions, respectively.

### Statistical analysis

The data are presented as the mean ± S.E.M. Statistical analyses were performed using a one-way ANOVA followed by Bonferroni's test. *P *values less than 0.05 were considered significant.

## Abbreviations

COX: cyclooxygenase; 

DMEM: Dulbecco's modified Eagle's medium; 

DRG: dorsal root ganglion; 

ERK: extracellular signal-regulated kinase; 

MAP: mitogen-activated protein; 

MEK: mitogen-activated protein kinase kinase; 

PKA: protein kinase A; 

PKC: protein kinase C; 

PLC: phospholipase C; SP: substance P.

## Competing interests

The author(s) declare that they have no competing interests.

## Authors' contributions

HBT participated in the design of the study, carried out all the experiments outlined in the manuscripts, performed the data analysis and wrote the manuscript. YSL designed and performed the immunocytochemical staining, and contributed to the analysis and interpretation of the data. KA designed the immunocytochemical staining, assisted with the data analysis and interpretation. YN participated in the design of the study, assisted with the data analysis and interpretation, and wrote the manuscript. All authors have read and approved the final manuscript.
